# History of Herbicide-Resistant Traits in Cotton in the U.S. and the Importance of Integrated Weed Management for Technology Stewardship

**DOI:** 10.3390/plants11091189

**Published:** 2022-04-28

**Authors:** Rohith Vulchi, Muthukumar Bagavathiannan, Scott A. Nolte

**Affiliations:** 1 AgriLife Extension, Department of Soil and Crop Sciences, Texas A&M University, College Station, TX 77843, USA; vulc18@tamu.edu; 2 AgriLife Research, Department of Soil and Crop Sciences, Texas A&M University, College Station, TX 77843, USA; m.bagavathiannan@ag.tamu.edu

**Keywords:** trait stacking, cotton, palmer amaranth, herbicide-resistant weeds, GM crops, tillage, cover crops, crop rotation

## Abstract

This paper reviews the history of herbicide-resistant (HR) traits in U.S. cotton since the beginning, highlighting the shortcomings of each trait over time that has led to the development of their successor and emphasizing the importance of integrated weed management (IWM) going forward to ensure their long-term sustainability. Introduction of glyphosate-resistant cropping systems has allowed for expansion of no-till systems more reliant on herbicides, favored less diverse crop rotations, and heavily relied on a single herbicide mode of action (MOA). With repeated applications of glyphosate over the years, biotypes of glyphosate-resistant (GR) *A. palmeri* and other weeds became economically damaging pests in cotton production systems throughout the U.S. Moreover, the reported cases of weeds resistant to different MOA across various parts of the United States has increased. The dicamba- (XtendFlex^®^) and 2,4-D-resistant (Enlist^®^) cotton traits (with stacks of glyphosate and glufosinate resistance) were introduced and have been highly adopted in the U.S. to manage HR weeds. Given the current rate of novel herbicide MOA discovery and increase in new HR weed cases, the future of sustainable weed management relies on an integrated approach that includes non-herbicidal methods with herbicides to ensure long-term success.

## 1. Economic Importance of Cotton to the U.S.

Cotton (*Gossypium hirsutum* L.) accounts for 25% of total fiber use globally [1] and is an important commercial crop in the U.S. in terms of both internal revenue and exports. Annual revenues generated from the cotton industry and allied services exceed USD 21 billion and provide employment to over 125,000 people [1]. Cotton is grown primarily for lint purposes; however, cotton seed contains 15–25% oil [2], and seed meal is also used as an animal feed. Cotton seed cake after oil extraction is a good organic fertilizer that contains 3.9% N, 1.8% P_2_O_5_, and 1.6% K_2_O on average [3]. Globally, the U.S. is the third largest producer and leading exporter of cotton, constituting around 35% of world exports [4], illustrating the significance of the U.S. cotton industry. An estimated total of 4.9 million hectares of cotton were planted in the U.S. in 2020, producing over 15 million bales of seed cotton and grossing over USD 4.7 billion [5]. A recent estimate shows that cotton provided a total economic impact of USD 18.5 billion across the entire U.S. economy during 2017–2019. This economic activity included USD 9.3 billion in gross domestic product (GDP) and USD 6.1 billion in labor income, supporting more than 130,600 workers. In Texas, where more than 40% of U.S. upland cotton is planted, 88% contained herbicide resistance traits [6], indicating widespread adoption of HR technology. 

## 2. History of HR Traits in Cotton

A total of 19 transgenic cotton events, including herbicide and/or insecticide resistance (HR/IR), have been approved for deregulation in the U.S. since 1994 [7]. Over the years, HR traits (Table 1) have been stacked with Bt-genes to offer broader pest management options and capture wider markets. The following sections describe the different HR cotton traits in more detail.

### 2.1. BXN™ Cotton (Bromoxynil-Resistant Cotton)

Bromoxynil-resistant cotton was first deregulated in the U.S. in 1994 and came to the market in 1995. It is the first transgenic HR trait in cotton that allowed for postemergence (POST) application of bromoxynil to control broadleaf weeds [8]. The transgene *nitrilase* from *Klebsiella pneumoniae* subsp. *ozaenae*, along with the 35S promoter and tml 3′ terminator, were used to generate two identical subunits of the *BXN* gene (*pBrx 74* and *pBrx 75*) that rapidly degrade bromoxynil in cotton plants. Bromoxynil is a photosystem II (PS II) inhibitor [9] and applications over the top of cotton provide growers with an additional option to control troublesome weeds during the cropping season. Before 1996, over-the-top (OTT) POST herbicide options were not available for use in cotton without potential interference with crop maturity or yield [10,11]. Commercialization of cotton with resistance to bromoxynil provided a great weed management option. However, because bromoxynil is not a broad-spectrum herbicide, it could not capture much of the market share and was phased out quickly. However, by introducing resistance into the crop and commercialization of bromoxynil-resistant cotton extended the market share for bromoxynil herbicide with little additional regulatory costs [12]. Therefore, research efforts were directed towards development of the Roundup Ready^®^ gene (Monsanto, St. Louis, MO, USA).

### 2.2. Roundup Ready^®^ Cotton (First-Generation GR Cotton)

After realizing the need for a broad-spectrum OTT POST herbicide for cotton, focus was placed on the development of transgenic cotton resistant to non-selective, broad-spectrum herbicides such as glyphosate. Before the introduction of GR crops, glyphosate was predominantly used in non-crop areas because of its non-selective nature. The ability to modify cotton with a glyphosate-insensitive gene allowed OTT use of glyphosate for selective weed management. Glyphosate has been described as the chemical of the century [13] and the ‘perfect herbicide’ yet devised, giving farmers one of the most efficacious weed management technologies in history [14].

Glyphosate inhibits the 5-enolpyruvylshikimate-3-phosphate synthase (EPSPS) enzyme, which catalyzes aromatic amino acid biosynthesis in the shikimate pathway [15]. Resistance to glyphosate is conferred through expression of the insensitive form of the *cp4-EPSPS* gene [16], which reduces the binding affinity of glyphosate [17]. The *cp4-EPSPS* gene was isolated from the soil bacterium *Agrobacterium* sp. strain *CP4*, originally discovered in a runoff sample at a glyphosate manufacturing site [18]. Over-expression of the sensitive target enzyme [19] and detoxification of the glyphosate molecule [20] were also explored for introducing glyphosate resistance in cell culture and whole plant systems of petunia (*Ruellia humilis* L.), tobacco (*Nicotiniana tobacum* L.), and carrot (*Daucus carota* L.), during the early days. However, these approaches failed to achieve commercially acceptable levels of resistance in cotton, and thus the insensitive *cp4-EPSPS* gene was used in the first-generation ‘GR cotton event 1445’ [21].

### 2.3. Sulfonylurea-Resistant Cotton

Sulfonylurea herbicides (SUs) and Pyrithiobac-sodium (Staple^®^ herbicide) control weeds by inhibiting the acetolactate synthase (ALS) enzyme that catalyzes the first common step in the biosynthesis of essential branched-chain amino acids isoleucine, leucine, and valine. Dupont tested different cotton lines with an *ALS* gene expressing a tolerant form of the ALS enzyme. The *ALS* gene in these cotton lines is a chimeric gene derived from two different tobacco *ALS* genes that both encode herbicide-sensitive versions of ALS [22]. Two resistance mutations (pro-Ala substitution at 191 position and Trp-Leu substitution at 568 position) were introduced into one of the *ALS* genes by in vitro site-directed mutagenesis. A DNA fragment containing the resistance mutations was moved into the second *ALS* gene by using a common restriction enzyme fragment. The gene introduced into this cotton line *19-51a* was a chimeric S4-HrA and encodes a resistant form of *ALS* attributable to two amino acid changes in the protein sequence [23]. However, this trait was not greatly adopted in the U.S. in part because of the widespread presence of *ALS*-inhibitor-resistant weeds and the success of GR cotton in the marketplace [24].

### 2.4. LibertyLink^®^ Cotton (Glufosinate-Resistant Cotton)

The Aventis company developed the cotton event ‘LLCotton25’ with resistance to the non-selective contact herbicide glufosinate-ammonium marketed under the trade name Liberty^®^. Glufosinate resistant *bar* (bialophos resistance) gene has been isolated from the bacteria *Streptomyces hygroscopicus*. The *bar* gene produces pat protein, which encodes for an enzyme phosphinothricin-N-acetyltransferase [25] that converts L-phosphinothricin to its inactive form through acetylation, thereby conferring resistance [26]. This cotton is marketed under the trade name LibertyLink^®^ (BASF, Florham Park, NJ, USA). Glufosinate is a competitive inhibitor of glutamine synthetase, the enzyme responsible for synthesizing glutamine from glutamate using ammonia as the substrate. Earlier, rapid death of plants treated with glufosinate were assumed due to absence of glutamine synthetase, leading to decline in glutamine content and accumulation of ammonia and, eventually, cell membrane disruption and death [27]. However, recent studies have proposed massive light-dependent generation of reactive oxygen species as the cause of glufosinate toxicity rather than ammonia accumulation [28]. Glufosinate is the only herbicide with this unique MOA and therefore can be an effective option for controlling GR weeds such as *A. palmeri*. Because glufosinate is a contact herbicide, weed control is highly dependent on spray coverage. Weed size at the time of application also has a substantial influence on the efficacy of this herbicide [29]. *A. palmeri* control with glufosinate is significantly reduced in plants taller than 8 cm [30,31]. Therefore, timely applications are required for optimal efficacy.

Low historic adoption rate of LibertyLink^®^ cotton varieties has been due to lack of transgene stack with glyphosate resistant trait, relatively poor agronomic performance of available varieties [32], and its relative ineffectiveness in controlling glyphosate-susceptible *A. palmeri* relative to glyphosate [33]. Until 2017, the only weed species with reported resistance to glufosinate in the U.S. were Italian ryegrass (*Lolium perenne* ssp. *multiflorum*) and annual bluegrass (*Poa annua* L.) [34,35]. However, populations of *A. palmeri* were recently found to survive multiple field applications of glufosinate in Arkansas [36]. Glufosinate resistance is not yet widespread, and therefore it can still be an effective option to control GR *A. palmeri* in cotton. 

### 2.5. Roundup Ready^®^ Flex Cotton (Second-Generation GR Cotton)

Roundup Ready^®^ cotton was rapidly adopted by U.S. cotton farmers and has been a significant part of U.S. cotton production after its market introduction. However, a constraint with the first-generation GR cotton was that OTT glyphosate applications were restricted to plants smaller than four true leaves. Due to the insufficient expression of the *cp4-EPSPS* gene driven by the weak promoter in male flower tissues, applications at/beyond the fifth true leaf stage required specialized spray equipment to aim the herbicide between the rows and away from the cotton plant, and any misapplication onto plants caused a fitness penalty and reduced yields [37]. This is because applications beyond four-leaf stage caused male sterility in the RR 1445 cotton event, wherein the pollen development is disrupted at the microspore stage at rates as low as 0.84 kg ae/ha [38]. To overcome this, Monsanto developed the second-generation GR cotton, the Roundup Ready^®^ Flex cotton event MON 88913 (Monsanto Co. St. Louis, MO, USA), which provided increased resistance to glyphosate through the reproductive phases of cotton growth and allowed OTT application of glyphosate until a week before harvest. The MON 88913 event was developed using the same gene and chloroplast targeting sequences as Roundup Ready^®^ cotton but has two copies of the *cp4-EPSPS* gene, with one of them under the regulation of P-FMV/TSF1 transcriptional promoter and the other under the regulation of P-35S/ACT8 transcriptional promoter. The presence of two copies of the *cp4-EPSPS* gene provided increased resistance to glyphosate during both vegetative and reproductive stages of plant growth [39]. Roundup Ready^®^ Flex cultivars were highly adopted after their commercial release; however, Roundup^®^ brand herbicides were the only glyphosate formulations approved for use OTT in these cultivars. 

### 2.6. GlyTol^®^ Cotton 

A few years later, Bayer (Bayer Crop Science LP, Research Triangle Park, NC, USA) developed their proprietary GR cotton in 2009 known as GlyTol^®^ cotton event ‘GHB 614’ that is similar to Roundup Ready^®^ Flex cultivars but with an alternative gene and promoter. GlyTol^®^ cotton was developed by transforming Coker 312 cv. with *2mEPSPS* gene by introducing site-directed mutagenesis into the wild-type *EPSPS* gene from maize (Thr—ile substitution at 102 position and pro—Ser substitution at 106 position) [40,41]. This modification conferred the protein a decreased binding affinity for glyphosate, allowing it to maintain sufficient enzymatic activity in the presence of the herbicide [42]. This event facilitated the use of any brand of glyphosate labelled for cotton. Widespread adoption of first- and second-generation Roundup Ready^®^ cotton and GlyTol^®^ cotton and resulting glyphosate-dependent weed control created high selection pressure on weeds such as *Amaranthus* spp. to evolve resistance to glyphosate. This situation created the necessity to stack multiple HR traits to control GR as well as susceptible weeds. 

### 2.7. GlyTol^®^-LibertyLink^®^ Cotton

Stacking HR traits in cotton started with the GlyTol^®^-LibertyLink^®^ event, commercialized in 2011. Cotton events ‘GHB 614’ and ‘LLCotton25’ were conventionally bred to express glyphosate resistance through *2mEPSPS* and glufosinate resistance through *bar* genes. Stacking genes provided the option to tank-mix glyphosate and glufosinate without crop safety issues and effectively controlled *A. palmeri* and other weeds, while decreasing the probability of resistance evolution. However, some field studies indicated that tank mixes of glyphosate and glufosinate were less effective at controlling *A. palmeri* than glyphosate applied alone. This indicated that sequential applications of these two herbicides were a better option for *A. palmeri* management, eliminating the benefit of tank-mixing herbicide for broad-spectrum weed control [43].

### 2.8. XtendFlex^®^ Cotton (Dicamba-Resistant Cotton)

With increased reports of GR weeds in cotton systems, necessity arose for an efficacious yet economic alternative for glyphosate. A group of researchers from the University of Nebraska at Lincoln (UNL) discovered and isolated a gene conferring resistance to dicamba from a soil bacterium that was successfully introduced into plant chromosomes providing up to 10-fold resistance to normal dicamba application rates [44]. UNL patented this technology and signed a licensing agreement with Monsanto to develop dicamba-resistant crops using the UNL technology.

Dicamba is converted to 3,6-dichlorosalicylic acid (DCSA) that lacks herbicidal activity by the three-component enzyme dicamba O-demethylase, isolated from the soil bacterium *Pseudomonas maltophilia* (strain DI-6). The three components include a monooxygenase, a reductase, and a ferredoxin, which serve as an electron transfer chain to transfer electrons from reduced NADH through the reductase to the ferredoxin and finally to the terminal component, the dicamba monooxygenase (dmo) [45]. Dicamba mimics plant growth hormones that stimulate cell elongation and differentiation, leading to rapid growth of stems, leaves, and petioles [46]. This abnormal plant growth disrupts cellular transport systems and eventually leads to the death of the plant. Susceptible plants exposed to even small quantities of dicamba show symptoms such as twisting and abnormal bending of branches and stem, necrosis of the meristematic tissues, and cupping of leaves [47]. Epinasty, which is downward bending of leaves and other plant parts resulting from excessive growth of the upper side, is another commonly observed symptom [48]. 

Bayer introduced dicamba resistance into cotton stacked with resistance to glufosinate and glyphosate. Dicamba and glufosinate resistance was introduced into the cotton event MON 88701 through *Agrobacterium*-mediated transformation of the cotton variety Coker 130 by inserting T-DNA containing both *dmo* and *bar* expression cassettes utilizing the vector PV-GHHT6997. After transformation, self-pollination and segregation were used to select those plants containing a single homozygous copy of the T-DNA, including both the *dmo* and *bar* expression cassettes, resulting in the selection of MON 88701. It was then combined through traditional breeding methods with GR cotton germplasm to deliver XtendFlex^®^ cotton [49]. 

Bollgard II^®^ XtendFlex^®^ cotton (Bayer Crop Science LP, Research Triangle Park, NC, USA) was the first triple stack HR cotton technology where dicamba could be used in preemergence (PRE) and POST applications until 7 days before harvest. This technology gave growers options to control GR *A. palmeri*, *A. tuberculatus*, and other HR weeds in cotton. Glufosinate can be an effective alternative to dicamba in mitigating drift issues and can be tank-mixed with residual herbicides to provide effective control of GR *A. palmeri* and other weeds in cotton systems [50,51]. However, stacked glufosinate resistance allows for broad spectrum weed control from emergence through early bloom growth stage only. This technology was commercialized in 2017 by Bayer Crop Sciences and available only in the U.S. A challenge with this technology is that dicamba can antagonize control of some grass species when applied in combination with graminicides or glyphosate [52]. Moreover, tank-mix combinations of dicamba and glufosinate ammonium are strictly prohibited by new dicamba formulation labels due to increased volatility [53], which compromises the advantage of tank-mixing these additional MOAs when treating GR weeds.

### 2.9. Enlist^®^ Cotton (2,4-D-Resistant Cotton)

Dow^®^ AgroSciences sought deregulation for the triple-stack Enlist^®^ cotton event in 2015. Enlist^®^ cotton provides resistance to the synthetic auxin herbicide 2,4-D, as well as to glyphosate and glufosinate. 2,4-D was the first synthetic herbicide to be commercially developed for controlling a wide spectrum of broadleaf weeds [54]. A transgenic cotton with 100-fold more tolerance to 2,4-D was obtained by introducing the *tfdA* gene from the bacterium *Alcaligenes eutrophus* [55]. The *tfdA* gene encodes the enzyme dioxygenase, which catalyzes the degradation of 2,4-D to the much less phytotoxic compound 2,4-dichlorophenol (2,4-DCP). Two genes encoding aryloxyalkanoate dioxygenase (AAD), AAD-1 (RdpA) from *Sphingobium herbicidivorans* and AAD-12 (SdpA) from *Delftia acidovorans*, were isolated, having 28 and 31% amino acid sequence identity to *tfdA*, respectively [56]. Both AADs can effectively degrade 2,4-D in that AAD-1 cleaves the aryloxyphenoxypropionate family of grass-active herbicides, while AAD-12 acts on pyridyl oxyacetate auxin herbicides [57]. An advantage of the Enlist^®^ weed management systems over the XtendFlex^®^ systems is the ability to tank-mix glufosinate and 2,4-D to control large *A. palmeri* [58], a potential tool for slowing the development of resistance. Although 2,4-D remains one of the most widely used herbicides globally, only isolated cases of resistant weed species have been reported because of its complex MOA. Multiple sites of action and the functional redundancy in the receptor family contributes to the low incidence of target site mutations for synthetic auxins. Consequently, only stacked mutations would render resistance to herbicides without an innate fitness cost [59].

### 2.10. Isoxaflutole-Resistant Cotton

The cotton event ‘GHB811’ was developed through *Agrobacterium*-mediated transformation of Coker 312 cv. with *HPPDPfW336-1Pa* and *2mEPSPS* expression cassettes by Bayer. The HPPD *PfW336-1Pa* gene encodes for the HPPD W336 protein that provides resistance to the HPPD-inhibiting herbicide isoxaflutole (IFT). The HPPDPfW336-1Pa coding sequence was developed by introducing a single point mutation (gly-Trp substitution at 336 position) to the wild-type HPPD gene derived from *Pseudomonas fluorescens*, a non-pathogenic bacterium that is ubiquitous in nature [60]. Expression of the HPPD W336 protein confers resistance to HPPD inhibitors, such as isoxaflutole, but the trait package is expected to have resistance to glyphosate, glufosinate, and dicamba also. BASF is also planning to commercial launch IFT cotton and is projected for 2023, with IFT being evaluated for use both as PRE and early POST applications across different locations in the U.S.

Isoxaflutole indirectly obstructs carotenoid biosynthesis, leading to bleaching of plant foliage followed by necrosis. Upon plant uptake, IFT is rapidly metabolized to the herbicidally active form diketonitrile (DKN; 2-cyclopropyl-3-(2-mesyl-4-trifluoromethylphenyl) -3-oxopropanenitrile) and is further metabolized to form a biologically inactive benzoic acid (2-mesyl-4-trifluoromethyl benzoic acid) [61]. Herbicide selectivity is achieved in tolerant, non-transgenic species by metabolizing DKN into benzoic acid more rapidly than sensitive species [62]. Isoxaflutole and DKN are both considered highly mobile in soil and have been studied as potential groundwater contaminants [63,64]. Currently, resistance to HPPD-inhibiting herbicides has evolved in biotypes of *A. palmeri* and *A. tuberculatus* in the U.S. Most HPPD-inhibitor-resistant *Amaranthus* spp. also have resistance to as many as four additional MOAs [24]. A recent survey found nearly 40% of screened *A. palmeri* populations contained survivors following mesotrione POST at 105 g ai ha^−1^, confirming HPPD-resistant *A. palmeri* in the southeastern U.S. [65]. A survey conducted in Texas found that 22% of the *A. palmeri* populations from the High Plains [66] and 38% of *A. tuberculatus* from Gulf Coast regions [67] were less sensitive to 93 g ai ha^−1^ tembotrione.

## 3. Benefits and Adoption of HR Cotton Traits 25 Years after Introduction

Benefits from HR crops since their introduction were categorized into agronomic, co-existence, health, yield, socio-economic, and environmental benefits globally [68]. The ability to manage weeds with less reliance on tillage, reduced soil erosion, improved soil and water conservation practices, lower CO_2_ emission, and less herbicide usage compared to conventional production systems can be attributed to HR traits. Introduction of resistance to glyphosate, glufosinate, dicamba, 2,4-D, and isoxaflutole into cotton facilitated the in-crop use of these non-selective herbicides, allowing for protection of yield [69]. An additional benefit of HR crops is the drastic reduction in injury from non-selective herbicides in cotton, better weed control resulting in higher income, and herbicide resistance management with alternative modes of action [70]. In a span of 21 years (1996–2016), an accumulated total of 340 million hectares of transgenic cotton (insect-resistant, herbicide resistant, or both) were grown commercially across the world [71]. The use of HT cotton globally delivered a gross farm income gain of about USD 130.1 million in 2016 alone, with a total gross farm income benefit of USD 1.92 billion (until 2016) since introduction. These farm income gains in cotton are mainly due to cost savings ranging up to 71% of the total gains, although there have been some yield gains of 27.47 million tonnes in other countries during the same period [72]. Without biotech crops, maintaining global production at 2016 levels would have required farmers to plant an additional 2.9 million ha of cotton [72].

After the first 22 years of commercialization of biotech crops, Brookes and Barfoot reported a cumulative total of USD 1.162 billion by the end of 2018 as economic benefits at farm level [73]. There are mixed opinions about the impacts of HR traits, but evidence clearly shows that these traits have generated benefit through reductions in fuel use, herbicide use, soil erosion, and consequently offering positive environmental impacts [74]. Evidence suggests a net reduction in herbicide active ingredient use by about 19.7 million kg between the years 1996–2016, representing a 6.3% reduction in usage, and in terms of the Environmental Impact Quotient (EIQ) indicator, an 8.3% net environmental improvement in the U.S. cotton systems [75]. However, it is also important to note that since the mid-2000s, the amount of herbicide active ingredient used on HT cotton in the U.S. has increased by 30% per hectare through a combination of additional usage of glyphosate in conjunction with increasing use of other herbicides. This shows that U.S. cotton farmers now make increasing use of additional herbicides with different MOA for managing glyphosate resistance in weeds [75].

**Figure 1:**
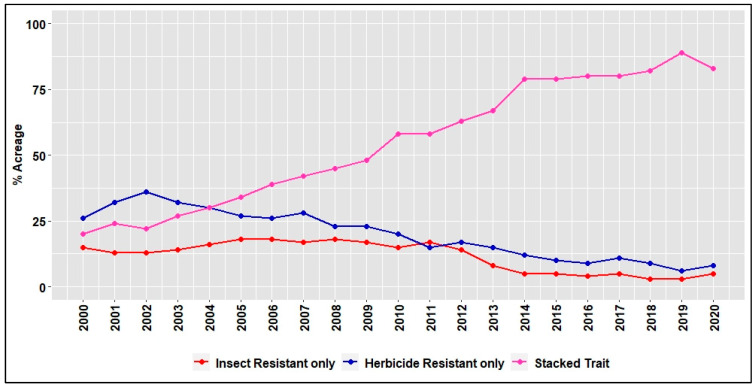
Adoption of genetically modified cotton in the U.S. during 2000–2020 [6].

**Figure 2:**
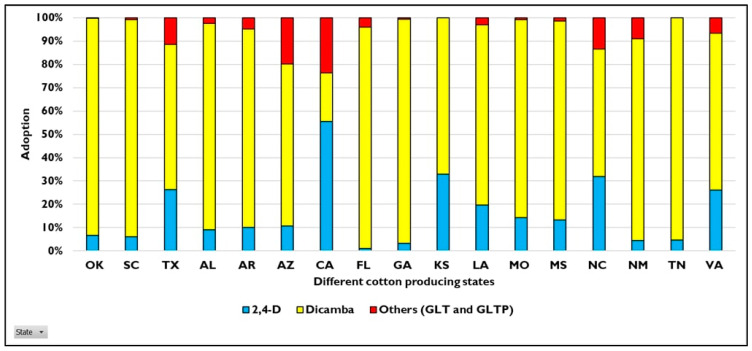
Adoption of auxinic herbicide-resistant cotton traits in different states in the U.S. during 2020 [76]. States not depicted in the graph have no data of cotton planting available. (2,4-D: Enlist^®^; Dicamba: XtendFlex^®^; GLT: GlyTol LibertyLink TwinLink; GLTP: GlyTol LibertyLink TwinLink^®^ Plus).

Increased adoption of stacked traits in cotton began during the mid-2000s with the onset of herbicide resistance and is currently adopted in more than 80% of the upland cotton planted in the U.S., conferring resistance to at least one herbicide (Figure 1) [6]. In the past 5 years, cotton traits conferring resistance to the glufosinate, dicamba, and 2,4-D herbicides have become the most widely adopted transgenic varieties (along with insect resistance), approved for cultivation and/or exports [71] (Figure 2).

The current U.S. cotton seed market is dominated by auxinic HR traits. During 2020, more than 90% of total upland cotton planted in the U.S. were resistant to auxinic herbicides such as dicamba and 2,4-D, with 73.3% acreage planted with dicamba-resistant cotton, and 19.51% with 2,4-D-resistant cotton [76,77] (Figure 2). Although there was a reduction in total area planted with upland cotton from 2019, adoption of both traits increased in 2020, indicating the value these traits provide to the grower [76,77,78].

## 4. HR Weeds in U.S. Cotton Production

**Figure 3:**
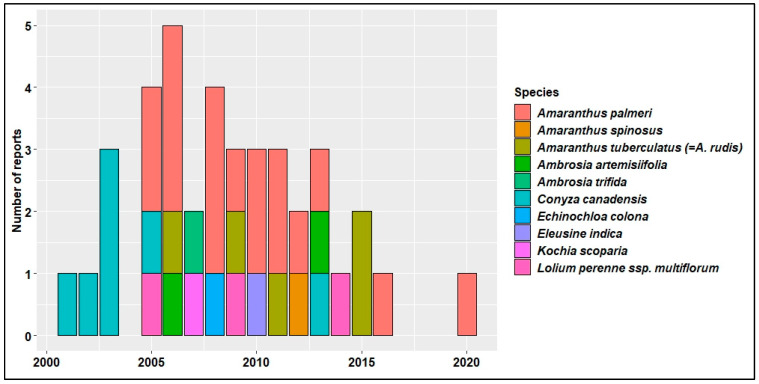
Annual reports of GR weed species in cotton since 2001 across the U.S. [24].

**Figure 4:**
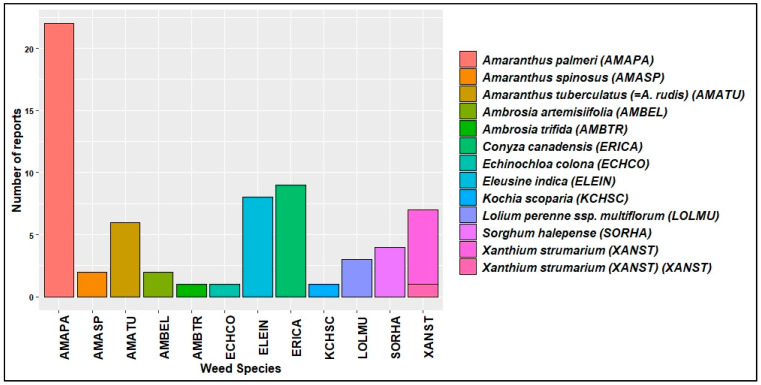
HR weed species reported across U.S. cotton production systems until 2021 [24].

Evolution and spread of HR weeds pose a major threat to these high value HR traits in the U.S. [79]. From 2005 to 2015, at least one case of new herbicide-resistant weed has been reported every year across the U.S. (Figure 3). Horseweed (*Conyza*/*Erigeron canadensis* (L.) Cronq) was the first to evolve resistance to glyphosate in U.S. cotton production systems [80]. As of December 2021, in the U.S. cotton production systems, 65 unique cases (species × site of action) of HR were reported in 12 different weed species (Figure 4), out of which a cumulative total of 39 cases of glyphosate resistance were reported in 10 weed species [24] (Figure 3). Of all the HR weeds, *A. palmeri* is arguably the most economically damaging weed in U.S. cotton production, with documented resistance to synthetic auxins, microtubule inhibitors, VLCFA inhibitors, EPSPS inhibitor, PPO inhibitors, and ALS inhibitors [24].

**Figure 5:**
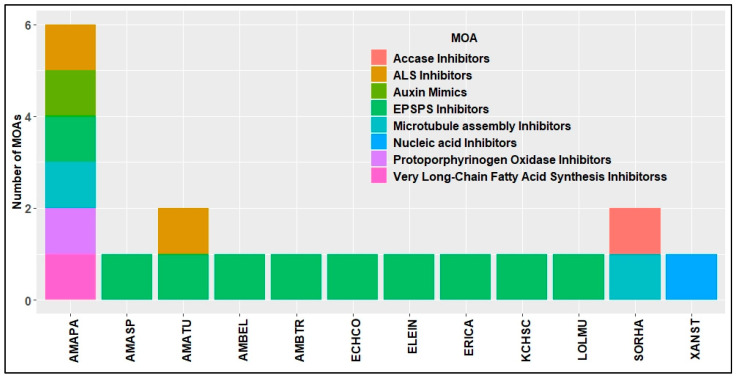
Weed species resistant to different MOAs in the U.S. cotton production systems [24].

Moreover, *A. palmeri* resistant to six different MOA in a single population has been reported in Arkansas [81]. In Texas, across different row crop production systems, *A. palmeri* evolved resistance to EPSPS, PSII, and ALS inhibitors [66]. No resistance has yet been reported in the tested populations for PPO inhibitors, HPPD inhibitors, or synthetic auxins in Texas. Glufosinate resistance has been confirmed in *A. palmeri* in Kansas and Arkansas, making it the first glufosinate-resistant broadleaf weed globally [36]. A susceptible *A. palmeri* population when exposed to sub-lethal doses of 2,4-D and dicamba over three generations increased LD_50_ by 2- and 2.5-fold, respectively [82]. In cotton production systems, 2,4-D and dicamba applications were not permitted during the cropping season until the commercialization of auxin-resistant transgenic crops in 2017. However, there is a long history of use of POST herbicides such as glufosinate and dicamba at lower rates in corn and sorghum as burndown applications in the field. Dicamba-resistant *A. palmeri* was confirmed in Kansas [83] and Tennessee [84] in 2019 and 2020, respectively, in long-term conservation tillage fields. *A. palmeri* movement through contaminated animal feed, manure, harvest equipment, and conservation seed plantings has been reported [85,86]. This demonstrates the possibility of populations of *A. palmeri* that evolved resistance to common herbicides between cotton and other crops can be moved into cotton systems through different routes. Herbicide-resistant *A. palmeri* populations were often overlooked in their early years of existence. Low levels of resistance in a population are often enough to cause economic loss [87]. Gene flow rates in *A. palmeri* are high, and a large proportion of the population can become resistant in just 2 years [85,87]. Biological qualities such as high fecundity, dioecious nature, and prolonged emergence enhance *A. palmeri*’s ability to adapt to selection pressure. Consequently, *Amaranthus* species have the highest incidence of herbicide resistance compared to other problematic weeds in the U.S. [24] (Figure 4). A total of 12 different weed species evolved resistance to 8 MOAs and on the basis of information available on gene flow rates possible in *A. palmeri*, this species must be considered a significant threat to new herbicides being brought to the market in the future (Figure 5).

Synthetic auxin technology is now being relied on for control of GR *A. palmeri* but is at high risk of loss if sound resistance management practices are not implemented. Tank mix combinations of glyphosate, glufosinate, and auxin-type herbicides can provide good control of GR weed populations [88,89] but may not be enough for effective control given the risk for non-target site resistance issues. Therefore, there is a need for HR management for *Amaranthus* species by proactive inclusion of diverse weed management strategies such as incorporation of multiple MOA into herbicide programs, crop rotations, and tillage to avoid evolution and spread of resistance. With small seeded broadleaf weeds evolving resistance to several POST herbicides in cotton rapidly, proper stewardship of existing technologies is of paramount importance. If stewarded properly, synthetic auxin herbicides (2,4-D and dicamba) can provide better management of *A. palmeri*, extend the viability of POST options. 

## 5. Non-Chemical Weed Control Options Available

**Figure 6:**
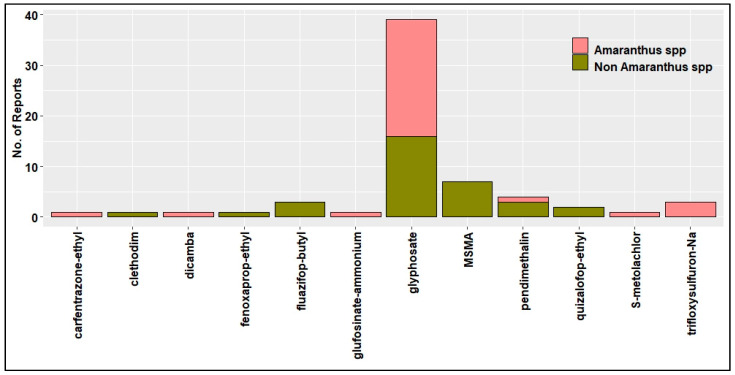
Reported cases of resistance to different herbicides in cotton production in the U.S. [24].

With the sustainability of chemical options looking ominous (Figure 6), non-chemical management practices such as tillage, crop rotation and cover crops have huge potential to reduce the burden of weed control on herbicides. These practices have negative impacts on seedbank persistence and weed seedling emergence dynamics when practiced over a period. Moreover, when combined with sound herbicide programs, they help effectively deplete the soil weed seedbank and reduce the risk of herbicide resistance. This could be particularly effective for managing weeds such as *A. palmeri* and *A. tuberculatus* with prolific seed production potential. 

### 5.1. Tillage Impacts on Weed Control

Tillage has long been used as a weed control tool within the cropping season, which can influence the longevity of weed seeds in the soil [90] depending on the species [91]. However, the area under conservation tillage has been increasing in the U.S. cotton production systems. Although conservation tillage practices are known to insure crop yields [92] and net returns [93] in dry land cotton production systems, particularly during low rainfall years, lack of soil inversion leads to the accumulation of weed seeds in the topsoil layer. For instance, in a study conducted to understand the influence of tillage on *A. tuberculatus* emergence and distribution, three times greater emergence was observed in no-till in comparison with chisel-till cultivation. Moreover, higher seedbank densities of 21 seed cm^−3^ at the 0–3 cm soil depth was found in a no-till system, compared to 10 seed cm^−3^ in chisel plowing [94]. The lack of weed seed burial in the no-till system favors the persistence of small-seeded annual weeds [95,96] that are able to emerge from a shallow soil depth compared to large-seeded weeds such as morning glory. Higher seedbank densities in the topsoil layer and a selection towards small-seeded annuals may subsequently lead to higher weed densities in no-till, compared to conventional till. Further, the absence of tillage favors perennial weeds (lack of disturbance to perennial underground structures) in conservation tillage [97,98].

Adoption of conservation tillage in the U.S. cotton systems is less than 30% in the southern Great Plains compared to more than 60% in the southeastern U.S. [99]; the southern Great plains region accounts for more than 40% of total cotton production. In the Texas high plains region, conventional till systems are still very popular, and shifting from conventional tillage to conservation tillage can influence weed population dynamics by altering the vertical distribution of weed seeds in soil and impacting weed seedbank persistence and seedling recruitment [100,101,102,103]. Although conservation tillage requires less capital equipment, prevents soil erosion, and improves water use efficiency and organic matter content in the soil, it may lead to more herbicide-dependent weed management. Conventional till systems, along with herbicide programs, could provide greater control of small, seeded annuals such as *A. palmeri* and *A. tuberculatus* by burying them deep into the soil profile (especially with deep tillage such as moldboard plowing), altering their emergence patterns and exhausting the seedbank [104]. However, with increased adoption of conservation tillage in cotton, adopting alternate tillage practices during different cropping seasons can be unconventional but an effective weed control option [105]. With these small-seeded annuals rapidly evolving resistance to most POST herbicides in recent years and severely impacting yield in cotton systems, long-term field studies testing the impacts of no-till vs. conventional till practices on long-term yield, seedbank replenishment, resistance evolution, and economic viability are necessary.

### 5.2. Cover Crops

Herbicides are one of the major expenses in the annual weed control costs in U.S. agriculture [106]. In the 2019–2020 National Cover Crop Survey report, the majority of respondents reported a cut in the herbicide costs and improved weed control with the use of cover crops in cotton [107]. Cotton is a slow-growing perennial and is highly sensitive to early season weed pressure. Flushes of *A. palmeri* that germinate late in the growing season or during the fallow period can significantly contribute to the soil seedbank and can become a big threat to cotton production in the long run [108]. Cover crops suppress weeds during the fallow season by altering the quantity of light reaching the soil surface [109], competing with weeds for space [110], altering the soil microclimate [111,112], and releasing allelochemical compounds [113,114,115]. Using cover crops reduces early season herbicide use, and when combined with reduced density of emerging weed populations at the time of spraying delay the probability of resistance [116].

Legumes, cereals, *Brassica* spp., and their mixtures were tested as cover crops to estimate their influence on early season and season-long weed control in cotton thus far. Legume cover crops provide nitrogen (N) credits to the subsequent cash crops [117] and consequently offer considerable savings on N fertilizers required to optimize cotton lint yields and improve soil quality [118]. The successful weed control achieved with legume cover crops is often attributed to biomass production, which can suppress weed germination and emergence. However, legume cover crops generally have low persistence on the soil surface due to a low C/N ratio [119]. On the other hand, cereal cover crops are known to produce high amounts of aboveground biomass, with cover crops such as cereal rye producing 20% to 30% of the total biomass belowground [120]. The high aboveground biomass production of cereal cover crops is also an excellent means of suppressing *A. palmeri* [121]. Winter wheat is a cheaper alternative to cereal rye with excellent weed control benefits. Another factor related to weed suppression provided by cereal cover crops is the release of allelochemicals produced by root exudates and plant residue decay that ultimately reduces seed germination. Brassica cover crops have the unique ability to produce glucosinolates, which are hydrolyzed to form a wide assortment of allelopathic isothiocyanates [122]. The amount of biomass produced by the cover crop is a great tool to estimate the achievable level of weed control. Biomass of around 4500 kgha^−1^ is necessary for adequate weed control [123], but the downside is reduction in cotton emergence due to high biomass amounts [124]. This could be particularly true when the residue interferes with adequate seed soil contact, leading to a negative impact on crop emergence [125]. The right herbicide options need to be selected for effective termination of cover crop mixtures, legume cover crops, and cereal cover crops [126]; the time of termination also plays an important role on weed control [127]. There are mixed reports on which would make the best cover crop option for biomass production, for example, single species vs. mixtures of different species in cotton [125,128]. The amount of total cover crop biomass production at spring planting is highly dependent on climate variables such as growing degree days and rainfall events. 

Planting cotton into live cover crops or terminating at planting for season-long weed control is still in its infancy. It has been reported that pigweed germination is phytochrome-dependent, and a low red to far-red ratio reaching the soil inhibits germination. A normal day light contains roughly equal proportion of red and far-red lights, but the red light is absorbed by the live covers. This reduces the red:far-red, leaving the phytochrome in its inactive form so that the seed will not germinate, thereby providing weed control during the early stages of cotton growth. However, care must be taken to prevent the delayed cover crop termination, which could result in a ‘green bridge’ of insect pests [127,129].

### 5.3. Future Directions for Cover Cropping

Cover crop species testing needs to be conducted in a wide range of environmental conditions for weed control and soil moisture retention, especially in dryland conditions where the majority of cotton is grown.

Better versions of mechanical burndown equipment such as roller crimper are required to completely terminate cover crops before planting to prevent competition from cover crops and improve cotton plant stands. 

Economic analyses need to be conducted to demonstrate the economic value of non-chemical weed control strategies for grower adoption. 

There is currently a dearth of peer-reviewed information regarding the adoption of cover crops throughout the U.S. Therefore, annual peer-reviewed surveys of cover crop adoption need to be conducted in various regions. 

Making growers their own scientists will allow them to develop weed control strategies for their specific farming situations seasonally using data from industry and local universities. 

Incentives such as carbon credits need to be established to promote conservation tillage practices. 

Constantly monitoring and researching the market drivers for adoption of cover crops helps effectively strategize the implementation of conservation practices.

### 5.4. Crop Rotation/Cropping Sequence Effects on Weed Population Dynamics

Cropping sequence is a dominant factor that influences species composition and seedbank [130]. Crop rotation is known to increase the diversity of weed communities, reducing the dominance of any single species in the long run by reducing their densities (Hume et al., 1991). This strategy could be particularly important when trying to manage weeds such as *A. palmeri*. Weeds that survive and produce seeds in one crop contribute to the seedbank from which weed seedlings are recruited in successive crops. Because of greater variability in the type and timing of soil, crop, and weed management practices, there are more opportunities for weed mortality events in rotations than in monoculture [131]. In a few studies where rotation effects on weeds have been examined without herbicides, rotation by itself led to reduced weed populations, especially when small grains were included in the rotation [132,133]. The effect of weed suppression by small grains can be attributed to allelopathy and increased exposure to predators and pathogens, especially for summer annual weed seeds that remain in the soil [132,133]. Herbicide rotation is defined as the application of herbicides of different MOA to multiple crops over multiple growing seasons in a field [134] and is the most common HR management strategy practiced by farmers [135]. Crop rotation also facilitates herbicide rotation with different MOA, thereby reducing the selection pressure on a single herbicide. Simulation models predicted that herbicide rotations or mixtures generally have the greatest effect in delaying resistance when the mechanism conferring resistance is target site-based, when the target weed species are highly self-pollinated, and when seed spread is limited [70]. The importance of diverse herbicide mixtures is growing as new crop technologies with resistance to multiple herbicides are being developed. However, positive cross-resistance, where resistance to one herbicide also confers some resistance to another, is likely to greatly reduce the usefulness of herbicide rotations, mixtures, and multiple-HR crop technologies as control measures, which is typical in weeds with non-target site resistance mechanisms.

### 5.5. Crop Rotation Roadblocks

Lack of rotational crop options for cotton that are economically attractive.

Impact of crop rotations on the fertilizer and pesticide industry, which favor monocultures, are unforeseeable.

Incorporating an alternative crop into the cropping schedule could influence the annual cotton production by replacing cotton acres, which could be a blow to local markets and exports.

Lack of research about the alternate crops that have competitive markets and storage infrastructure. 

Role of climate change in picking a rotational crop has been understudied. 

Subsidies, financial incentives, and federal crop insurance programs that exist for cotton products could influence the adoption of crop rotation. 

Federal policies influence adoption of rotation (if there is a ‘cotton independence’ act tomorrow, it would need a ramp up in cotton production throughout the U.S, consequently making it harder to incorporate a rotational crop in schedule). 

There is a need for the private players to step up and develop business models to incorporate crop rotation into the herbicide trait technology package.

## 6. Conclusions

Small seeded broadleaf weeds such as *A. palmeri* are rapidly evolving resistance to several POST herbicides in cotton. Tillage, cover crops, and crop rotations are proven non-chemical strategies to control *A. palmeri*, and it is important to reduce selection pressure by herbicides with these alternative strategies. Studies were conducted previously, testing herbicide programs in different cover crops and tillage systems [136,137] and different herbicides in crop rotations in cotton [138]. These non-chemical options not only provide in-season weed control but also affect weed infestations during fallow periods and exhaust the seedbank. While the current method of using residual herbicides in herbicide programs provides in-season control, supplementing them with non-chemical options discussed here would also provide off-season management of *A. palmeri*. More IWM practices need to be developed and evaluated for long-term weed control, as well as economic and environmental sustainability of U.S. cotton production systems.

## Figures and Tables

**Table 1:** Chronological order of deregulation of different HR traits in cotton in the U.S.

Trait Name	Transgene (s)	Herbicide(s) Resistant to	MOA	Company	Year Deregulated
BXN	*nitrilase*	Bromoxynil	PS-II inhibitor	Calgene	1994
Roundup Ready^®^	*Cp4-EPSPS*	Glyphosate during vegetative phase only	EPSPS inhibitor	Monsanto	1995
Sulfonylurea-resistant cotton	Mutant form of *Acetolactate synthase* (ALS)	Pyrithiobac	ALS inhibitor	DuPont	1995
LibertyLink^®^	*Bar*	Glufosinate	Glutamine synthetase inhibitor	Aventis	2003
Roundup Ready^®^ Flex	2 *cp4-EPSPS* genes	Glyphosate during both vegetative and reproductive stage	EPSPS inhibitor	Monsanto	2004
GlyTol^®^	*2m-EPSPS*			Bayer CropScience	2009
XtendFlex^®^	*dmo, EPSPS*, *bar*	Dicamba, glyphosate, and glufosinate	Synthetic auxin, EPSPS, and glutamine synthetase inhibitors	Monsanto	2015
Enlist^®^	*tfdA*, *EPSPS*, *bar*	2,4-D, glyphosate, and glufosinate	Synthetic auxin, EPSPS, and glutamine synthetase inhibitors	Dow Agro-Sciences	2015
IFT	*HPPDPfW336-1Pa*, *2mEPSPS*	Isoxaflutole	HPPD inhibitor	Bayer CropScience	2018

(PS-II: photosystem-II; EPSPS: 5-enolpyruvylshikimate-3-phosphate synthase; bar: bialaphos resistant; dmo: dicamba monooxygenase; 2,4-D: 2,4-dichlorophenoxyacetic acid; IFT: isoxaflutole; HPPD: 4-hydroxyphenylpyruvate dioxygenase).
